# *Trypanosoma vivax* elicits both Th1 and Th2 immunological responses in experimentally infected cattle

**DOI:** 10.1371/journal.pone.0329459

**Published:** 2025-07-31

**Authors:** Cristina Cholota-Iza, Marbel Torres-Arias, María Augusta Chávez-Larrea, Fausto Bedoya-Paez, Mishell Cisneros-Ruiz, Georgina Morales-Moreno, Jorge Ron-Román, Claude Saegerman, Armando Reyna-Bello

**Affiliations:** 1 Maestría en Biomedicina, Facultad de Ciencias de la Salud, Universidad Internacional SEK, Quito, Pichincha, Ecuador; 2 Grupo de Investigación en Sanidad Animal y Humana (GISAH), Carrera de Ingeniería en Biotecnología, Departamento de Ciencias de la Vida y de la Agricultura, Universidad de las Fuerzas Armadas ESPE, Sangolquí, Pichincha, Ecuador; 3 Research Unit of Epidemiology and Risk Analysis applied to Veterinary Sciences (UREAR-ULg), Fundamental and Applied Research for Animal and Health (FARAH) Center, Department of Infections and Parasitic Diseases, Faculty of Veterinary Medicine, University of Liege, Liege, Belgium; 4 Grupo de Investigación en Sanidad Animal y Humana (GISAH), Carrera de Ingeniería Agropecuaria, Departamento de Ciencias de la Vida y la Agricultura, Universidad de las Fuerzas Armadas ESPE, Sangolquí, Pichincha, Ecuador; Kerman University of Medical Sciences, IRAN, ISLAMIC REPUBLIC OF

## Abstract

Bovine trypanosomosis caused by *Trypanosoma vivax* is a health problem of economic importance in South America. In Ecuador, the presence of *T. vivax* was first reported in 2018; however, the isolates found in Ecuador are still being studied, mainly on issues related to virulence, pathogenicity, and immune response. To this end, this study aimed to evaluate the cellular and humoral adaptive immune response *in vivo* in experimentally infected cattle with *T. vivax*. The study lasted 42 days (with samples collected twice weekly) and was conducted in two cattle experimentally infected with an isolate of *T. vivax* circulating in Ecuador (TvET1) and two uninfected cattle as controls. Parasitemia was determined by the Brener method and relative gene expression (RGE) of six cytokines was evaluated by RT-qPCR to determine the Th1 response (IFN-γ, TNF-α, IL-1β, IL-12) and the Th2 response (IL-4 and IL-10). Additionally, the total IgG and the IgG1 (Th2) and IgG2 (Th1) subclasses levels were measured using an in-house iELISA. During the study, the animals exhibited four parasitemia peaks concomitant with the cytokines IFN-γ and IL-10. These cytokines, like TNF-α, showed a significant RGE increase (p < 0.05) in infected animals. The presence of total IgG, IgG1 and IgG2 was significant (p < 0.05) in infected animals, and presented a solid monotonic relationship over time. The predominant immunoglobulin subclass was IgG1, and we found that this response was similar to the total IgG. The present study allowed us to highlight the Th response of cattle to *T. vivax* infection, which is polarized into both a Th1 and a Th2 response. This information contributes to understanding the host-pathogen interaction with strains circulating in Ecuador. The thoroughness of our study can provide the needed knowledge to develop new diagnostic tests and even possible alternatives for vaccine development.

## Introduction

Bovine trypanosomiasis caused by *Trypanosoma vivax* is a significant health problem in livestock in tropical and subtropical regions of Latin America, Africa, and Asia [1]. In South America, the presence of *T. vivax* has been reported in most countries, with the exception of Chile and Uruguay [2]. In Ecuador, *T. vivax* was first identified in 2018 following an outbreak in cattle in the Manabí province in the Coastal Region [3]. In the Americas, *T. vivax* is transmitted exclusively by mechanical means via hematophagous vectors, such as flies of the families Tabanidae and Musidae [4,5].

*T. vivax*, like other members of the salivarian trypanosome group, is a protozoan that typically circulates in the host’s blood [6]. However, evidence of *T. vivax* DNA has also been found in tissues such as the spleen, skin (ear tip), brain, liver, epididymis, testis, and uterus [7]. Infection with this protozoan in livestock is primarily characterized by fever, anemia, weight loss, and problems with the nervous and reproductive systems [8]. These symptoms and related problems reduce animal productivity, thereby limiting the socioeconomic development of the livestock sector in endemic regions and negatively impacting the food security of the population [9,10].

Studies show that *T. vivax*, like other trypanosomatid species, has developed mechanisms for immune evasion that allow it to survive within the mammalian host, primarily through strategies involving variable surface glycoproteins (VSGs) [11]. Antigenic variation of VSGs, along with other VSG-associated mechanisms such as controlled endocytosis of the VSG-antibody complex, constitutes the primary mechanisms by which trypanosomes evade the host immune system [12,13]. In addition, the immunosuppressive environment created by trypanosomes and the depletion of memory B cells also disrupt the immune system’s balance in favor of the parasite [14].

The host immune response to trypanosome species can vary depending on the parasite strain, the host’s age, genetics and health status, which is reflected in the host’s breed [15]. Salivarian trypanosomes are extracellular parasites, so the dominant adaptive immune response should be the humoral response mediated by T helper 2 (Th2) cells [16]. However, studies show that the response can also be cellular, demonstrating a high presence of pro-inflammatory cytokines produced by T helper 1 (Th1) cells [11].

Polarization toward a Th1 or Th2 immune response is defined by the cytokine profile secreted by CD4 + Th lymphocytes, through activation of the proteins signal transducers and activators of transcription (STAT) 4 and STAT6, respectively [17]. Proinflammatory cytokines such as IFN-γ, IL-1β, IL-12, and TNF-α are characteristic of a Th1 response, whereas anti-inflammatory cytokines such as IL-4, IL-5, IL-10, and IL-13 are characteristic of a Th2 response [18]. Furthermore, in animals, a Th2 cytokine profile generally stimulates IgG1 secretion by B cells, whereas a Th1 cytokine profile promotes IgG2 secretion [19–21].

The host response to a pathogen in natural and experimental infections may differ due to environmental factors or animal genetics [22]. In natural infections, hosts are exposed to a variety of doses and routes of entry, resulting in a more heterogeneous immune response that is representative of the typical clinical scenario [23]. In contrast, experimental infections frequently utilize an inoculum with known concentrations, artificial routes, and meticulously controlled environmental conditions. These conditions can elicit a more homogeneous and predictable immune response [24].

In experimental infections with *T. vivax* in cattle and buffalo, a high presence of IFN-γ and TNF-α has been demonstrated [24,25], while in natural infections of cattle with *T. vivax*, the presence of IL-10 has also been detected, in addition to TNF-α [26]. Regarding the presence of antibodies in animals infected with *T. vivax*, the analysis of IgG subclasses in the serum of experimentally and naturally infected animals has shown higher levels of IgG1 compared to IgG2 [27]. However, activation of the adaptive immune response isn’t sufficient to eliminate the parasite from infected animals [22].

The study of the immune response elicited by *T. vivax* in cattle is crucial for understanding the host-pathogen interaction and for generating in-depth knowledge of these immune mechanisms. Although a Th1 or Th2 response is commonly observed in infections with intracellular and extracellular microorganisms, respectively, parasites have also been shown to elicit a mixed Th1/Th2 response [28].

The presence of a mixed response, although it may confer protection to the host, may also activate a deficient non-typical immune response, thereby evading the protective response [29,30]. In contrast, certain extracellular parasites elicit a Th2 response in the host. However, this response does not offer protection due to the immunomodulatory effects of parasite-secreted substances. Instead, a Th1 response appears to be associated with enhanced host resistance to infection [31].

It is acknowledged that, to date, no vaccine has been developed to prevent infection by *T. vivax* Given the nature of the immune response elicited by a strain of *T. vivax* circulating in Ecuador, it is possible to continue studying the parasite molecules that elicit this immune response and determine whether it confers protection to the host. This information can be used to identify potential vaccine candidates and formulations that can further stimulate the protective response (Th1 or Th2). Consequently, these studies can serve as a valuable source of biomarker findings (e.g., cytokines and antibodies) that can serve as targets for the development of diagnostic tests.

This information will provide essential tools for searching, developing, and researching serological diagnostic tests, vaccines, and effective treatments to control this hemoparasite. This will improve diagnosis, treatment, and prevention, reduce prevalence and economic losses, and ensure food safety and security. Few studies have been conducted on the immune response of cattle to *T. vivax*; therefore, the present study aimed to evaluate the adaptive humoral and cellular immune responses in cattle experimentally infected with a *T. vivax* isolate circulating in Ecuador*.*

## Materials and methods

### Trial design and obtaining biological samples

Four male Holstein cattle between 4 and 6 months of age from nearby areas were used for this study between August and September 2022 in the integrated farm of the Carrera de Agropecuaria IASA I of the Universidad de las Fuerzas Armadas ESPE. Animals were confirmed negative for *Anaplasma marginale*, *Trypanosoma* spp. and *Babesia* spp. by molecular diagnosis of these hemotropic pathogens using previously described protocols [3,32–34]. Animals were randomly assigned to two blocks of two individuals each. Two steers in one block were inoculated in the jugular vein with ${1x10}^{6}\;$trypanomastigotes in 1 mL/animal of a *T. vivax* isolate TvET1, obtained from Napo Province, Ecuador. The animals in this block are called “infected” and designated IB1 and IB2. The animals in the uninoculated block are called “control” and designated CBs.

For animal welfare reasons, the infected animals were treated with Isometamidium Chloride Hydrochloride (Hemoveex, Laboratorios REVEEX) at the end of the study.

The veterinary clinical trial (VCT) lasted 42 days, during which we collected blood samples (both with EDTA anticoagulant and without anticoagulant) from each animal at 13 points: day 0 (D0), D4, D7, D11, D14, D18, D21, D25, D29, D32, D35, D40, and D42. D0 was the day we inoculated the cattle with the parasites. We processed the blood samples with anticoagulant to determine 1) the parasitemia of the infected animals and 2) the relative gene expression of cytokines using RT-qPCR. We processed the blood samples without anticoagulant to determine: 1) the presence of total IgG and 2) the subclasses IgG1 and IgG2.

During the trial, the animals remained in two isolated and sealed pens as a biosecurity measure to prevent the spread of the hemoflagellate in the area and to avoid possible infection of the calves with other diseases. The diet consisted of water at *libitum*, 1.3 kg of concentrate, and 1 kg of hay per steer. The VCT protocol was approved by the Animal Ethics Committee of the Universidad San Francisco de Quito, as reported under “OFFICE: 2021-009”, according to the principles of the 3Rs (Replacement, Reduction, and Refinement).

### Determination of parasitemia

We determined the parasitemia on the sampled day each from four animals experimentally infected with *T. vivax* using the fresh drop method described by Brener [35].

### Determination of cytokine gene expression by RT-qPCR

#### Peripheral blood mononuclear cells (PBMC) isolation and RNA extraction.

We isolated peripheral blood mononuclear cells (PBMC) from the anticoagulant-treated blood samples of the four steers included in the study (infected and controls) by density gradient centrifugation using Lymphoprep™ (Stemcell Technologies, Cat: 07851). Briefly, 4 mL of fresh blood was mixed with an equal volume of PBS, layered over 4 mL of Lymphoprep™, and centrifuged at 800 g for 1 hour. The resulting PBMC ring was collected and quantified using a hemocytometer with 0.4% Trypan Blue (Gibco, Cat: 15250–061), as described by Strober (2015). A volume containing ${6x10}^{6}$ cells was centrifuged at 10,000 g for 10 minutes to obtain a PBMC pellet, which we resuspended in 250 µL of PBS. We then extracted RNA from the resuspended PBMC using TRIzol™ (Invitrogen). The quantity and quality of the obtained RNA were determined by spectrophotometry on a NanoDrop™ 2000 (Thermo Fisher Scientific), and integrity was assessed by 1.5% agarose gel electrophoresis. We considered RNA samples with 230/260 ratios of 1.8–2.1 and 280/260 ratios of 1.8–2 and with intact strands for further processing. Finally, RNA samples were treated with DNase I (Invitrogen™, Cat: 18068015) to eliminate any residual genomic DNA. We performed the RNA extraction and DNase treatment procedures according to the manufacturer’s instructions.

#### Gene expression by RT-qPCR.

To determine the cytokines gene expression involved in the Th1 and Th2 responses, we analyzed the RNA samples from the cattle using a two-step RT-qPCR. First, we performed RNA reverse transcription (RT) using SuperScript™ III reverse transcriptase (Invitrogen™, Cat: 18080044) and oligo (dT) (Invitrogen™, Cat: AM5730G), according to the manufacturer’s instructions. At the end of the processes, the cDNA of each sample was quantified by spectrophotometry.

We performed qPCR assays on the cDNA to amplify the genes expressing the cytokines IFN-γ, IL-1β, IL-4, IL-10, IL-12, and TNF-α, as well as the reference genes β-actin, GAPDH, and HPRT. Table 1 shows the details of the primers used to amplify the nine genes and references, except for IL-12, which was designed using the Primer Blast tool.

**Table 1:** Primer information used in RT-qPCR.

Genes	Primer Sequence (5’-3’)	Size (bp)	AT (°C)	Reference
**TNF-α**	F: TCTTCTCAAGCCTCAAGTAACAAGC R: CCATGAGGGCATTGGCATAC	103	59	[36]
**IL-1β**	F: GCTCTTGGGGTAGACTTTGGGGT R: CCCGAGCCCTGCTGATCCAT	143	64	[36]
**IFN-γ**	F: ATTCAAATTCCGGTGGATGA R: TTCTCTTCCGCTTTCTGAGG	109	57	[37]
**IL-12**	F: GCTGAGGAGAGCCTGCTTATT R: TCTTGGGTGGGTCTGGTTTG	115	58	Designed
**IL-10**	R: CCTTGTCGGAAATGATCCAG F: CGCAGGGTCTTCAGCTTCT	115	56	[37]
**IL-4**	F: GCTGAACATCCTCACAACGA R: CGCCTAAGCTCAATTCCAAC	123	58	[37]
**β-actin**	F: CATCGGCAATGAGCGGTTC R: GTGTTGGCGTAGAGGTCCTT	144	60	[37]
**GAPDH**	F: GGGTCATCATCTCTGCACCT R: GGTCATAAGTCCCTCCACGA	176	59	[37]
**HPRT**	F: TTGCCGACCTGTTGGATTAC R: TTGACCAAGGCAAGCAAAGT	176	59	[38]

5’-3’, primer sequence direction; F, primer forward direction; R, primer reverse direction; bp, base pair; °C, Celsius grade; AT, Annealing temperature.

The reaction mixture was prepared with 1X GoTaq(R) qPCR Master Mix (Promega, Cat: A6001), 0.3 µM of each primer, and 200 ng of cDNA. In order to PCR optimization, we added 5% DMSO to the reaction mixture for TNF-α gene amplification. We performed the qPCR assay on a C1000 Touch Thermal Cycler equipped with the CFX96 Touch Real-Time PCR Detection System (BIO-RAD). The thermocycler conditions were an initial denaturation cycle at 95°C for 2 min, 30 cycles of 15 s at 95°C for denaturation, 30 s at the optimal annealing temperature for each primer pair (Table 1), and 30 s of extension at 60°C; followed by a final extension cycle at 60°C for 5 min. We analyzed each sample in duplicate and obtained Ct (cycle threshold) data from the CFX96 master system.

We analyzed the gene expression of the different cytokines by a relative expression analysis normalized to reference genes. To select the best reference gene, we calculated the coefficient of variation using the Ct data of the three reference genes tested. The β-actin gene had the lowest coefficient of variation, so we used its the coefficient of variation data to determine the relative gene expression (RGE). We calculated the RGE ratio using the method developed by Pfaffl (2001), which accounts for differences in primer efficiency. We used the average Ct of D0 for each group of animals (infected and controls) as the control Ct.

### Determination of the kinetics of total IgG, IgG1, and IgG2 by indirect ELISA

We analyzed the presence of total IgG (IgGt) and subclasses IgG1 and IgG2 in bovine serum using an in-house optimized iELISA. We started coating the ELISA plates (Thermo Scientific, Cat No. 442404) with 100 μL of the antigen at 2 μg/mL, diluted in carbonate-bicarbonate buffer at pH 9.6, and incubated overnight at 4°C. The antigen used was a recombinant truncated paraflagellar rod protein (PRFLr) from a local *T. vivax* isolate produced at the Animal Biotechnology Laboratory of the Universidad de las Fuerzas Armadas ESPE, according to the protocol of Bedoya-Páez et al., 2024 (paper in preparation). We performed the blocking step with 200 μL of 5% skim milk in PBS buffer. Then, 100 μL of each serum was added, diluted 1/400 for IgGt, and 1/100 for IgG1 and IgG2 (the serum of each animal was analyzed in duplicate). 100 μL of secondary antibody was added at a dilution of 1/20000 for anti-bovine IgG HRP (Sigma-Aldrich, Cat: A5295) and 1/10000 for anti-bovine IgG1 HRP (Bethyl Laboratories, Cat: A10116P) and anti-bovine IgG2 HRP (Bethyl Laboratories, Cat: A10117P), respectively. After adding each solution (blocking solution, serum, and secondary antibodies), the plates were incubated at 37°C for 1 hour and then washed. The plate was washed 3 times after incubation with the antigen and blocking solution and 5 times after incubation with the serum and secondary antibodies. Serum and secondary antibody dilutions were prepared in PBS-Tween 0.1%, and the wash solution was 150 mM NaCl with 0.1% Tween.

We added 50 μL of TMB Liquid Substrate (MP Biomedicals, Cat: 152346) and incubated for 30 minutes for the development. Finally, we read the plates using a Multiskan SkyHigh Microplate Spectrophotometer (Thermo Scientific) at 450 nm.

The standard deviations of the optical density (OD) data from the negative control serum were used to obtain the cutoff point (CP) for each in-house ELISA and to determine each infected animal’s seroconversion day.

### Data analysis

Statistical analyses were performed using GraphPad Prism 8 and R Studio. The RGE ratio data and iELISA OD values from the two control animals were averaged (at each sampling time point) and then used as the mean (CM) in subsequent work. For the infected animals (IB1 and IB2), parasitemia and RGE results were analyzed individually. In the case of the iELISA OD results, the average of the two animals was used due to the similarity of the OD values at each time point. All data sets were analyzed for normality using the Shapiro-Wilk test. To compare the RGE and iELISA OD results between control and infected animals, a paired analysis (at each time point) was performed, using the paired t-test for data following a Gaussian distribution and the Wilcoxon rank-sum test for non-normally distributed data. Correlation analysis was performed using the Pearson correlation coefficient “r” (for parametric data) and the Spearman correlation coefficient “rs” (for non-parametic data). Finally, the Granger causality test was applied to data showing a moderate or strong correlation with parasitemia.

## Results

### Determination of parasitemia

**Fig 1:**
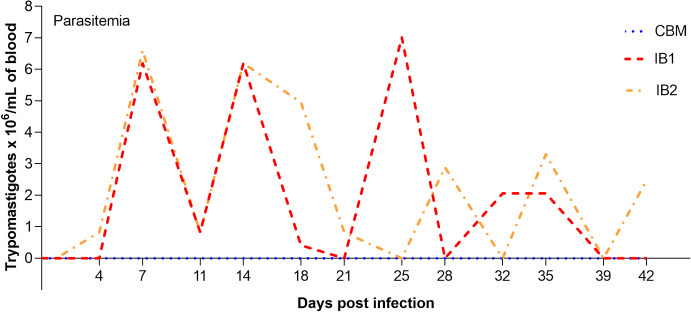
Parasitemia of steer IB1 and IB2 experimentally infected with *T. vivax.* Parasite Parasite load in Trypomastigotes x 10^6^/mL of blood during 42 days post-infection (red and orange lines). Control bovines (blue line).

In Fig 1, we can see that parasites are already present in IB2 on day 4 post-infection, although the first peak of parasitemia is observed on day 7 in both animals (IB1 and IB2). Throughout the VCT period, the animals exhibit four peaks of parasitemia, with the first two occurring approximately every seven days and the other two taking a little longer (10–15 days). It is observed that the parasite load begins to decrease after the first two parasitemia peaks in IB2 and after the third peak in IB1. The highest recorded parasite load (7.01 x 10^6 trypomastigotes/mL of blood) was observed around day 25 in animal IB1. However, this is not significantly different from the observed parasitemia in the first two peaks in both animals. As expected, no parasitemia was shown in the controls.

### Analysis and findings for gene expression kinetics of cytokines

**Fig 2:**
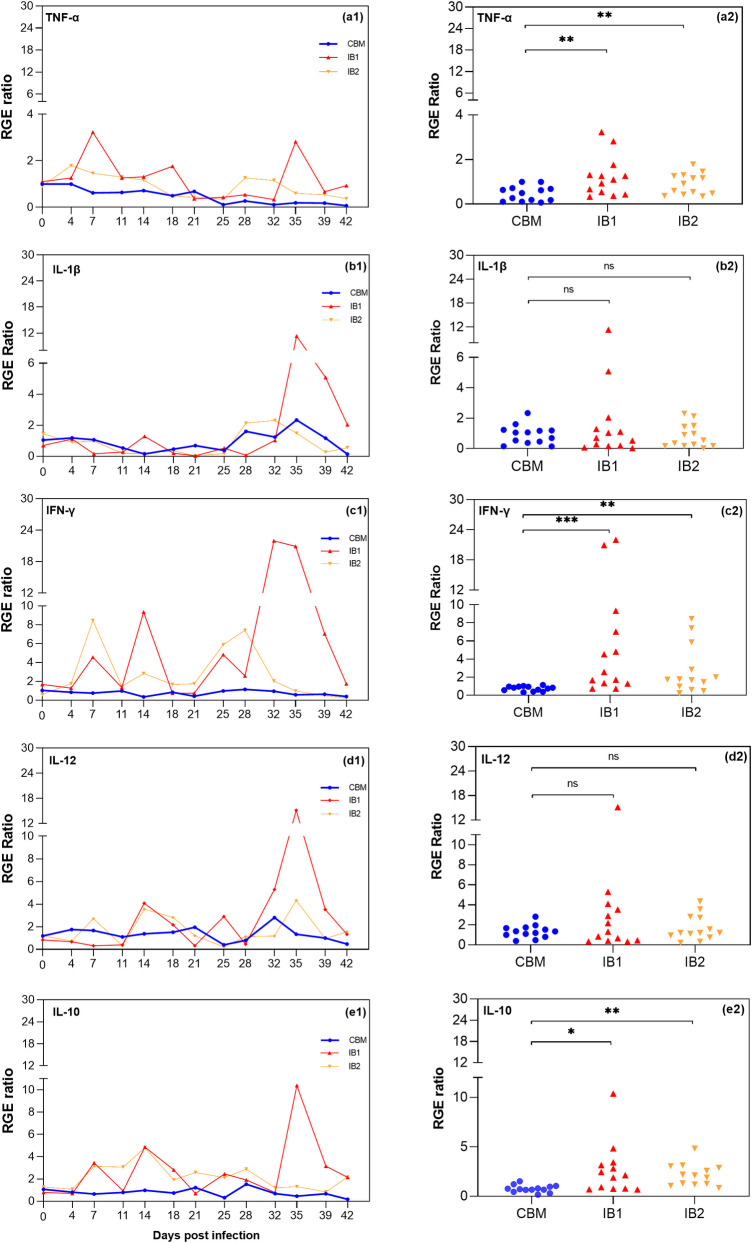
Kinetics of relative gene expression (RGE) over the 42-day duration of the study and scatter plot of cytokines’ relative gene expression (RGE) ratios at 13 analyzed time points. Pro-infPro-inflammatory cytokines: a) TNF-α, b) IL-1β, c) IFN-γ, and d) IL-12. Anti-inflammatory cytokines: e) IL-10. Part 1. CBM (blue line): kinetics of the RGE ratio mean of the control animals. Red line (IB1) and orange line (IB2): kinetics of the RGE ratios of the infected animals. Part 2. Blue spheres: RGE ratios mean of the control animals (CBM) for each cytokine. Red triangles: RGE ratios of IB1 for each cytokine. Orange inverted triangles: RGE ratios of IB2 for each cytokine. Paired statistical analysis for non-Gaussian distributed data using the Wilcoxon rank-sum test: TNF-α (CBM vs IB1, p = 0.0024), IL-1β (CBM vs IB1, p = 0.9319), IFN-γ (CBM vs IB1, p = 0.0005 and CBM vs IB2, p = 0.0044), IL-12 (CBM vs IB1, ρ=0.2439), IL-10 (CBM vs IB1, p = 0.0171). Paired statistical analysis for Gaussian distributed data using the Paired t-test: TNF-α (CBM vs IB2, p = 0.0023), IL-1β (CBM vs IB2, p = 0.6321), IL-12 (CBM vs IB2, p = 0.3565) and IL-10 (CBM vs IB2, p = 0.0005).

The kinetics of gene expression for the five analyzed cytokines (IFN-γ, IL-1β, IL-4, IL-10, IL-12, and TNF-α) are shown in Figs 2a1–2e1. In general, an increase in the RGE of the pro-inflammatory cytokines IFN-γ and TNF-α, as well as the anti-inflammatory cytokine IL-10, was observed in infected animals (IB1 and IB2) throughout the infection (Figs 2a1, 2c1, and 2e1). A paired statistical analysis of the data collected on different sampling days revealed a significantly higher RGE of these cytokines in infected animals compared to controls (p < 0.05) (Figs 2a2, 2c2, and 2e2). The RGE of the other analyzed cytokines, IL-1β and IL-12, did not show statistically significant changes compared to controls (Figs 2b2 and 2d2). For IL-4, the cycle threshold (Ct) results were delayed (>32 cycles), and most of the analyzed samples did not exceed the threshold within 35 cycles of qPCR. Therefore, these results are not shown.

Upon analyzing the RGE between the *T. vivax-*infected animals, it is observed that one of the cattle, IB1, exhibits a higher overexpression of the IFN-γ, TNF-α, and IL-10 genes. Despite this, the RGE of these three cytokines is significant in both animals (Figs 2a2, 2c2, and 2e2). This indicates that the infected cattle do not respond with the same intensity and show some variation in kinetics over the infection period. However, both animals respond to the *T. vivax* infection with a similar trend.

Both infected animals show two distinct peaks of TNF-α RGE in the early and late days of infection (Fig 2a1). However, the RGE decreases slightly as the days after infection progress. Among the three cytokines that showed significant overexpression in the infected animals, TNF-α was the lowest, reaching peak expression levels of only ~3 and ~2-fold higher than the controls in IB1 and IB2, respectively. In contrast, the cytokines IFN-γ and IL-10 displayed four expression peaks approximately every 7 days, with the highest peak occurring between D32 and D35 (Fig 2c1). IL-10 reached up to 10- and 5-fold higher expression levels in IB1 and IB2, respectively. The cytokine that showed the most significant increase in expression in the infected cattle compared to the controls was IFN-γ, reaching up to ~22-fold and 8.5-fold higher in IB1 and IB2, respectively.

Regarding the pro-inflammatory cytokines IL-1β and IL-12, although the paired analysis demonstrates no significant difference between infected and control cattle, an elevated expression peak was observed around D35 in IB1 (Figs 2b1 and 2d1). This day also corresponds to the highest expression peak of the cytokines IFN-γ and IL-10.

### Presence of IgGt, IgG1 and IgG2 antibodies

The optimized in-house iELISAs allowed the detection of anti-PRFLr of *T. vivax* IgGt, IgG1, and IgG2 in the infected animals. The antibody response was similar in both infected animals, in contrast to cytokine expression. The response intensity was higher in IB1 than in IB2. Therefore, the data were analyzed using the mean OD of these two bovines (IBM).

**Fig 3:**
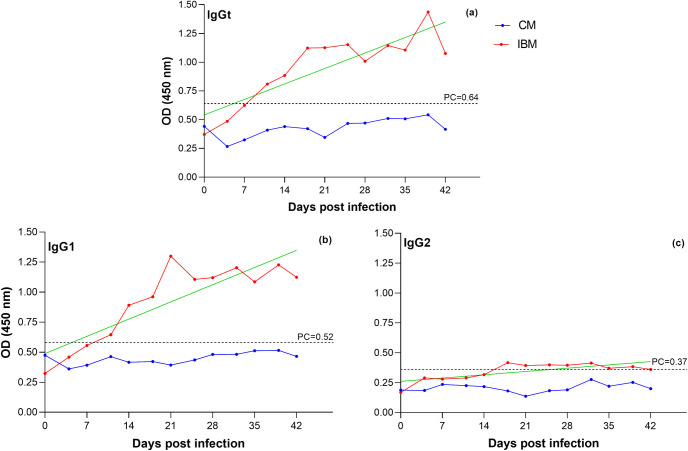
Kinetics of IgGt, IGg1 and IgG2 antibodies post inoculation of *T. vivax* during 42 days postinfection. Red lines (I Red lines (IBM): antibody kinetics based on the mean OD of the iELISA of the infected animals of each antibody analyzed. Blue lines (CM): antibody kinetics based on the mean OD of the iELISA of the control animals of each antibody analyzed. PC: Cut-off point of the iELISA test for each antibody analyzed.

According to the iELISA cut-off point (PC) for each antibody analyzed (IgGt, IgG1, and IgG2), the infected animals were considered seropositive for *T. vivax* after day 7 for IgGt and IgG1 (Figs 3a and 3b). At the same time, seropositivity for IgG2 was observed after day 14 (Fig 3c). However, in the iELISA for IgG2, the mean OD of the seropositive serum did not deviate significantly from the cut-off point (Fig 3c).

Observing the kinetics of the analyzed antibodies (Fig 3), it becomes evident that immunoglobulins increase with the days after parasite inoculation in cattle. This is confirmed by the analysis of the Pearson (r) or Spearman (rs) correlation coefficient (depending on the data distribution) of the mean OD over time: IgGt shows a strong positive correlation (r = 0.88), IgG1 a strong positive correlation (r = 0.90) and IgG2 (rs = 0.79) a robust positive monotonic correlation.

**Fig 4:**
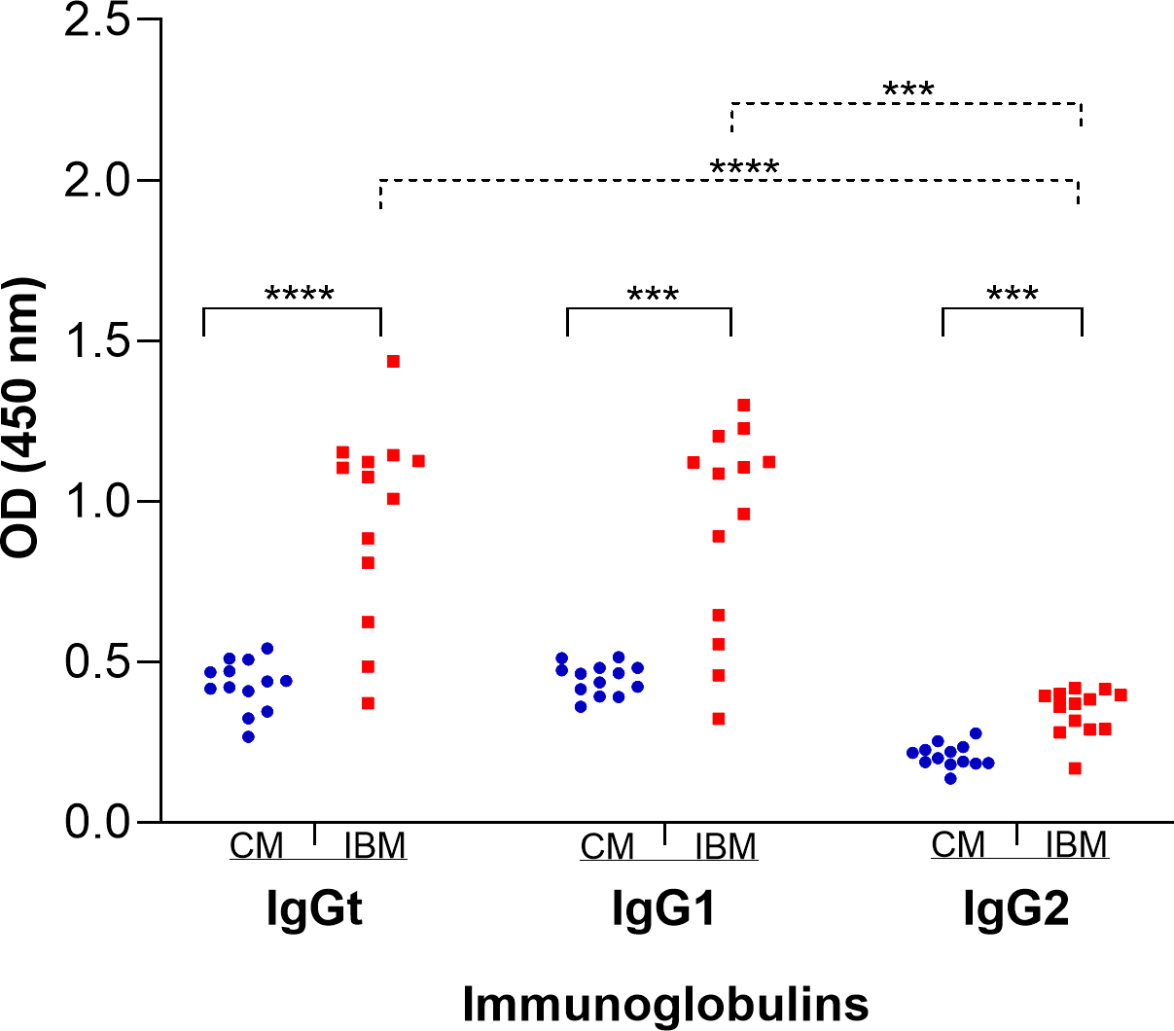
Scatter plot of iELISA OD values to detect the presence of antibodies, IgGt, IgG1 and IgG2 at 13 analyzed time points. Blue spheres and red squares: OD means of the control animals (CM) and infected animals (IBM), respectively. Paired statistical analysis for Gaussian distributed data using the Paired t-test: IgGt (CM vs IBM, p < 0.0001), IgG1 (CM vs IBM, p = 0.0001) and IBM (IgGt vs IgG1, p = 0, 4076). Paired statistical analysis for non-Gaussian distributed data using the Wilcoxon rank-sum test: IgG2 (CM vs IBM, p = 0.0005), IBM (IgGt vs IgG2, p = 0.0002) and IBM (IgG1 vs IgG2, p = 0.0002).

As the infection progresses in the animals, the antibodies increase exponentially, with IgGt and IgG2 rising until around day 18 (Fig 3a and 3c) and IgG1 until day 21 (Fig 3a). After this point, their levels remain relatively constant, although with slight peaks of increase. The presence of all three anti-*T. vivax* immunoglobulins analyzed were significantly higher (p < 0.05) in the infected animals compared to the controls (Fig 4). When analyzing the presence of antibodies only in the infected animals, no significant difference was observed between IgGt and IgG1 (p > 0.05). However, IgG2 was significantly lower than IgGt and IgG1 (p < 0.05), suggesting that the detected IgGt was mainly due to IgG1.

### Cytokine and antibody behavior about parasitemia

**Fig 5:**
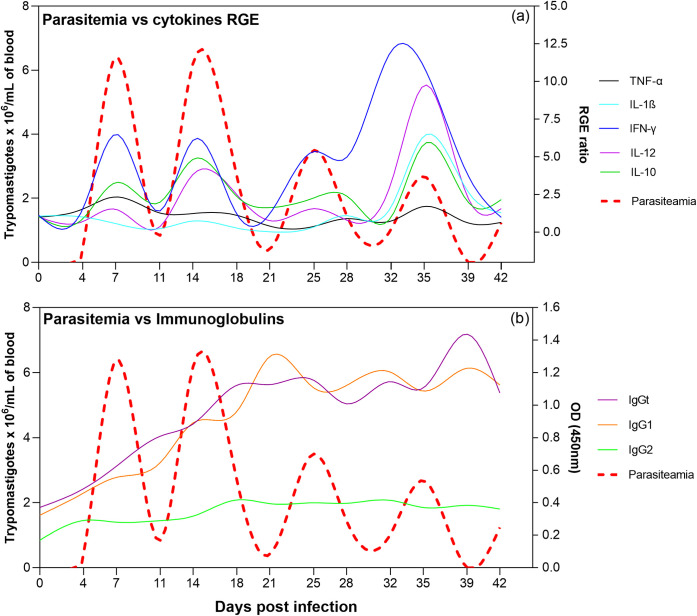
Curves illustrate the behavior of relative gene expression (RGE) of cytokines and immunoglobulins in response to parasitemia peaks in the infected animals. a) Curves sha) Curves showing relative gene expression (RGE) behavior based on parasitemia over the 42 days of infection. b) Curves showing the behavior of immunoglobulins based on parasitemia over the 42 days of infection.

Finally, the behavior of cytokines and immunoglobulins in the infected animals was evaluated about the increase or decrease in parasitemia levels over the 42-day study period. As seen in Fig 5, cytokine expression seems to be concomitantly related to parasitemia levels, but not to the presence of immunoglobulins.

When analyzing the Spearman correlation coefficient for the three cytokines that showed significantly higher RGE in the infected animals (Fig 2), TNF-α exhibited a weak positive monotonic correlation (r = 0.32), IFN-γ a modest positive monotonic correlation (rs = 0.50), and IL-10 a robust positive monotonic correlation (rs = 0.79). IL-10 was the cytokine that showed the highest correlation with parasitemia. Furthermore, the Granger Causality Test revealed that IL-10 was the only cytokine statistically proven (p < 0.05) caused by parasitemia.

Although IL-1β and IL-12 did not show a statistically significant increase in expression (Fig 2), they exhibited elevated RGE peaks during the last parasitemia peak, coinciding with the lowest parasitemia level throughout the study period (Fig 5a). This pattern is also observed for the cytokines IFN-γ and IL-10.

Although the presence of immunoglobulins (IgGt, IgG1, and IgG2) did not exhibit concomitant fluctuations with parasite load (rs = 0.01, rs = −0.15, and rs = 0.15, respectively), a slight increase in antibodies was observed after each parasitemia peak during the latent phase (Fig 5b).

## Discussion

This study aimed to elucidate information on the adaptive immune response (Th1 and Th2) in cattle infected with a *T. vivax* isolate (TvET1) circulating in Ecuador. Despite advances in recent years, studies on *T. vivax* remain limited, especially those related to the immune response of cattle infected with local strains of *T. vivax*. Given that the host’s immune response to pathogen infection can vary depending on the host’s defense capacity, as well as the pathogenicity and virulence of the strain [39], it is essential to continuously conduct studies on the immune response with different isolates of *T. vivax*.

The parasitemia dynamics in the animals during the VCT were as expected, considering that the appearance of parasitemia peaks at regular intervals is a ubiquitous feature in infections with *Trypanosoma* sp. [40,41], conferred by the VSG antigenic variation present in this parasite type [42]. However, although the two infected animals showed identical parasitic waves in the first weeks, a shift in the curve was observed later in one of the animals. This difference can be attributed to host heterogeneity in the defense response to the infection. It is now recognized that host heterogeneity, among other factors, plays a fundamental role in the evolution of virulence and transmission rates [43]. Host heterogeneity can arise for various reasons, including infection history, general physiological state, and genetic variation [44].

It is a widely accepted that genetic variation in both the host and the parasite is critical for the evolution of infectious diseases, as it affects host defense response, parasite transmission, and virulence [45,46]. Consequently, virulence, transmission rate, reproduction, and recovery rate have been reported to be highly dependent on parasite load [43,47]. The parasitic load of the TvET1 isolate observed in the animals in this study is high (~7 x 10^6 trypomastigotes/mL of blood), considering that average reported in other studies, which range from 0.021 x 10^6–13 x 10^6 parasites/mL of blood [41,48,49]. Parasite load and other factors can affect the cattle’s ability to mount an effective immune response [46].

The most effective host immune response against pathogens is the adaptive response, which is activated when the innate response is insufficient to clear infection, and pathogens persist in the host [50]. CD4 + Th cells (Th1, Th2, or Th17) are the leading players in the adaptive response. Once activated, they secrete specific cytokines that activate other immune cells, including B cells, which proliferate and secrete specific antibodies that help to eliminate pathogens [51]. The time required for the adaptive immune response to activate and become effective can take one to two weeks [52]. In our study, the animals’ adaptive response was activated as early as the first week, as evidenced by the significant expression of cytokines (IFN-γ, TNF-α, and IL-10) and antibody seroconversion (IgG1 and IgG2).

Studies of the host immune response to infection with *Trypanosoma* species (*T. brucei* or *T. congolense*) state that the adaptive response begins with major histocompatibility complex (MHC)-II-mediated antigen presentation to Th lymphocytes [53]. The response then polarizes into Th1 and Th2 responses, with pro-inflammatory cytokines, such as IFN-γ and TNF-α, and anti-inflammatory cytokines, such as IL-10, predominant [11].

Similarly, in bovine infections with *T. vivax*, animals polarize both Th1 and Th2 responses according to reported cytokine profiles. In an experimental infection study in Brazil, although a different dynamics of cytokine expression were reported across the three animals studied, the cytokines that showed at least one peak in expression level in at least one animal within the first 42 days of the trial were IL-1β, IL-4, IL-6, IL-10, IL-12, TNF-α, and IFN-γ [24]. In contrast, only IL-10 and TNF-α were reported in a study of natural infections in Africa [26].

The cytokine expression results in our study are similar to those described in previous studies, as significantly higher expression of IFN-γ, TNF-α, and IL-10 was observed. Likewise, although the expression of IL-1β and IL-12 was not significant when analyzing the kinetics over the 42-day period, both showed at least one peak of increased expression within this time frame. In the case of IL-4, which was undetectable in the two infected animals, this is consistent with previous reports from natural infections where IL-4 was not detected [26], and in one of the animals from an experimental infection where no increase in IL-4 expression was observed within the 42 days of infection [24].

Of the three cytokines that showed increased expression during the 42 days of infection, the peaks in IFN-γ expression were the highest. It is a well-established fact that IFN-γ is the main cytokine synthesized by CD4 + Th1 lymphocytes [54], as well as by other cells such as NK cells, NKT cells, and γδ T lymphocytes [55,56]. Studies have identified γδ T lymphocytes as an essential cell population in ruminants, involved in both innate and adaptive immune responses, with a high density in PBMC, representing up to approximately 60% in young animals [55,57,58]. Bassi et al. [48] reported that animals infected with *T. vivax* showed a high population of γδ T lymphocytes and CD4 + cells during the parasitemia peaks. In our study, the abundant expression of IFN-γ in animals infected with *T. vivax* may be attributed to synthesis by CD4 + Th1 lymphocytes and γδ T lymphocytes.

During the adaptive immune response, IFN-γ and TNF-α, among other cytokines, are synthesized by CD4 + Th1 cells, a subpopulation of lymphocytes characteristic of a cellular response. This type of response is generally activated when pathogens are intracellular (Tolomeo & Cascio, 2024) or located in extravascular tissues, as can occur in *T. brucei* infections [59]. Although *T. vivax* circulates freely in the blood, there have been cases where *T. vivax* DNA has been found in extravascular tissues [7]. This could explain the activation of a cellular response in the infected animals of this study, as the proinflammatory cytokines IFN-γ and TNF-α were detected. On the other hand, activation of the adaptive immune response, specifically antigen presentation of antigens in the case of *T. vivax*, must occur in the spleen, as this parasite circulates mainly in the blood [60].

TNF-α, along with reactive nitrogen intermediates (RNI) and reactive oxygen intermediates (ROI), are trypanocidal factors produced by macrophages, which are activated by the presence of IFN-γ to fight infection [61]. TNF-α has long been considered a trypanocidal agent; for example, a study demonstrated that mice lacking this cytokine were more susceptible to *T. vivax* [62]. TNF-α in the blood, produced by monocytes, and in tissues, produced by macrophages, contribute to the first line of defense against trypanosome infection [63].

The cytokines produced by CD4 + Th cells fight infection and regulate inflammation, a necessary process to relieve and balance the stress generated [22]. IL-10 is an anti-inflammatory cytokine (part of the cytokine profile of a Th2 response) that contributes to the immunosuppression of inflammation generated mainly by monocytes or macrophages [64]. In trypanosome infections, the presence of this cytokine has been reported [65], influencing the reduction of proinflammatory cytokines such as TNF-α [22]. This would explain the slight decrease in TNF-α expression as the infection progressed and the increase in IL-10 in infected animals.

An exciting aspect of this study is the simultaneous correlation between cytokine expression, mainly IFN-γ and IL-10, and parasitemia. The presence of parasite peaks in the animals can trigger the activation and clonal expansion of CD4 + Th cells [48], which are responsible for the immediate production of proinflammatory and anti-inflammatory cytokines, necessary to combat infection at each peak of antigenically distinct trypanosomes. In addition to the simultaneous correlation of parasitemia with cytokine expression as the disease progressed, an increase in expression was also observed at the last parasitemia peak, which decreased as the infection advanced. This may suggest that as the infection progresses, the animals generate B and T cells of memory capable of enhancing the defense response against *T. vivax*, thereby reducing the intensity of parasite proliferation and enhancing the immune response through the cytokine surge expressed.

This can be caused by two main factors. The first one is the level of VSG gene variation. Studies have shown that *T. vivax* has only about 150 VSG genes and pseudogenes [66,67], while, *T. brucei*, for example has about 1500 genes and pseudogenes [68,69]. Moreover, these VSGs are not presented in a mosaic form (with one VSG variant expressed at a time) as in other trypanosomes, but instead in an antigenic VSG profile that appears to be closely related and co-dominantly expressed, with some VSGs appearing in later peaks [70]. The second one is the percentage of non-variable membrane protein coverage. In *T. vivax,* only about 50% of the parasite surface is covered by VSGs, in contrast to other trypanosome species, where up to 98% of the parasite surface is covered by VSGs [71]. Both the limited genetic variability of the VSGs (fewer genes and pseudogenes, as well as repetitive VSGs) and the higher percentage of non-variable proteins could contribute to the presence of an immunological memory response against these repetitive and non-variable proteins in subsequent waves of parasitemia.

On the other hand, it is known that the cytokine profile produced by CD4 + lymphocyte subpopulations, mainly led by IFN-γ and IL-4, acts during the S phase of B cell division (phase during which the genomic DNA inside the nucleus is duplicated), directing the *ch* gene rearrangement and resulting in the IgG2 and IgG1 subclasses, respectively [72]. Our results demonstrate the presence of both IgG subclasses, although IgG2 is present at a lower level. The presence of IgG2 indicates the effect of IFN-γ in IgG subclass switching. However, the more significant proportion of IgG is of the IgG1 subclass, despite the absence of IL-4.

According to studies [73], the switch to IgG2a is IFN-γ dependent, whereas the presence of IgG1 is not entirely dependent on IL-4. Although IL-4 enhances the IgG1 found in cells exposed to VSG, switching to IgG1 still occurs in the absence of IL-4, albeit at lower levels.

The absence of detectable IL-4 expression in this study, despite the predominance of the IgG1 isotype, may reflect the transient and low-abundance nature of this cytokine during immune responses to protozoan infections. Previous research has shown that IL-4 exhibits a narrow window of expression. In fact, it is estimated that IL-4 has a half-life of 45 min, this short duration is compensated by an effect called short-term memory, which allows Th2 cells to re-transcribe *IL-4* mRNA for about 3 days due to an epigenetic effect that keeps the cells ready to express again if stimulated by the antigen [74]. In this sense, it is possible that as parasitemia decreases, IL-4 expression also decreases, making it undetectable in our assay for monitoring its kinetics.

Although both IgG subclasses analyzed showed reactivity against *T. vivax* infection, the IgG1 subclass was dominant. These results are consistent with other studies in cattle showing that the presence of IgG1 in both natural infections (African strains) and experimental infections (South American strains) with *T. vivax* [27]. Similarly, in a study on mice testing the IFX antigen (flagellar protein subunit of IFX from *T. vivax*) as a vaccine candidate, the antibody isotyping generated against IFX was entirely of the IgG1 subclass [75].

In contrast to the cytokine expression, which coincides with parasitemia peaks, antibodies against *T. vivax* are continuous after reaching the latent phase, despite the slight decrease in parasitemia peaks and their increase when parasitemia declines. This could be caused by a high availability of antigens and an elevated formation of immune complexes (Ag-Ab), which make antibodies less detectable by an immunological test [76]. The antibody production by plasma cells against a specific antigen (Ag) can remain in the host’s blood for weeks [77]. In our study, throughout the 42 days of the experimental infection, the analyzed immunoglobulins G (IgGs) did not decrease precisely because of the continuous immunological stimulus exerted by parasitemia.

In this research and others conducted by Gkeka et al. [78], there is evidence of an antibody (Ab) immune response to *Trypanosoma* sp. infections, primarily of the IgG1 subclass. Still, it appears that these antibodies cannot effectively combat the infection, or at least, not alone. A study by Autheman et al. [75], showed that IgG1 antibodies against an invariant protein subunit of *T. vivax* (IFX) passively inoculated into rodents, did not confer protection against exposure to the live parasite. However, information on the functions of IgG subclasses in mice revealed that IgG1 does not effectively recruit immune effector functions, such as complement activation or binding to high-affinity activating Fc receptors [79]. Based on this information, Autheman and collaborators [75] generated IgG2 antibodies in the laboratory by mutations in IgG1. Subsequently, IgG2 was passively inoculated into rodents. The results revealed that the IgG2 subclass provided excellent protection against parasite exposure. Clearly, the presence of proinflammatory and anti-inflammatory cytokines, as well as IgG1 and IgG2 antibody subclasses, suggests that the adaptive immune response of cattle against *T. vivax* polarizes a Th1/Th2 response. While this may contribute to a beneficial balance between the two responses to control parasite proliferation, stimulation a Th1 response could be a mechanism by which *T. vivax* evades the host immune system. It has been observed that this may occur due to an imbalance in STAT4 and STAT6 signaling. For example, recent studies [80] have shown that SARS-CoV-2 and the monkeypox virus cause dysregulation of STAT4 and STAT6 expression, with STAT6 being predominantly activated, inducing a Th2 response. This, in turn, inhibits the Th1 response, which is essential for viral clearance, and promotes immune evasion. In our study, something similar may be happening when *T. vivax* triggers a dominant activation of STAT4, leading to a Th1 response (typical of intracellular microorganisms and associated with the presence of IFN-γ) and partially inhibiting the Th2 response (typical of extracellular microorganisms and associated with the presence of IL-4).

This study has been a significant step forward in elucidating some aspects of the host-pathogen interaction with a *T. vivax* isolate in Ecuador. Since *T. vivax* is an extracellular parasite (Gonzatti et al., 2014), it was expected that the dominant immune response would be a Th2 response with IL-4 presence; curiously, despite the abundance of IgG1, the expression of this cytokine was absent.

The study of cytokine expression profiles and the presence of immunoglobulin G subtypes has frequently been utilized to elucidate the type of response that a pathogen elicits in the host [81–83]. Despite the limited number of animals in the study, the RT-qPCR results clearly indicate a significant increase in both Th1 and Th2 cytokines. Furthermore, ELISAs revealed the substantial presence of IgG subtypes, indicative of a Th1 and Th2 response.

As mentioned in the introduction, the type of response (Th1 or Th2) can be determined by the presence of specific cytokine profiles or IgG subtypes. In the present study, two distinct techniques were employed to analyze the two types of molecules that define the immune response. The results obtained from these analyses were consistent. In summary, animals infected with TvET1 *T. vivax* isolate exhibit molecules indicative of both Th1 and Th2 responses.

In order to enhance the study’s statistical power, the intrinsic variables of the experimental animals were standardized, including species, breed, gender, and age. The study also sought to mitigate the potential impact of confounding variables, including health status, management practices, and feeding methods. As Festing [84] asserts, to enhance the statistical reliability to 80% in studies involving experimental animals, particularly laboratory animals, it is recommended that the minimum number of infected animals be set at four, with an optimal range of 10–20 animals. Furthermore, the aforementioned assertions pertain to studies encompassing multiple treatments, a scenario that does not apply to the present study. Other studies with similar research criteria have used a small number of animals in their experiment [40,41,85,86]. However, it is important to note that, because of the challenges of managing a large number of calves, the results presented in this work should be regarded as an approximation of the real results.

In a nation of limited logistical, economic, and technological resources, such as Ecuador, it was deemed expedient to infect and observe a total of four animals, in addition to incorporating two control animals. However, given the pioneering nature of this experimental infection in Ecuador, priority was accorded to aspects related to handling, feeding, clinical follow-up, biosecurity of the work environment, and ethical considerations, as delineated in [84]. As indicated by the factors previously cited, the immune response may be influenced by these elements in terms of its magnitude and balance [87]. However, the results obtained in the present study demonstrate a consistency in the tendency of the biomarkers analyzed (IgG and cytokines), although one of the subjects exhibited a higher relative expression of cytokines.

The findings have indicated the presence of strain-specific variability in virulence and pathogenicity among certain strains of *T. vivax* that are currently in circulation within the South American region [41]. The present study focused on the study of the multiplicative capacity of the isolate TvET1 and the modulation of the host response to infection with a local strain of *T. vivax*.

The finding that the TvET1 *T. vivax* isolate elicits a Th1 and Th2 response in cattle, although in a limited number of experimental animals, clarifies the type of polarized response of the isolate. Furthermore, this finding paves the way for future research endeavors aimed at identifying antigens that can effectively stimulate an immune response capable of counteracting the parasite. This research may also facilitate the development of a vaccine.

Conversely, the analysis of the biomarkers revealed a notable presence of IgG1, which may serve as a potential target for serological diagnostic tests. It is imperative that studies be conducted to analyze these molecules in animals at the field level. Additionally, robustness tests, such as sensitivity and specificity tests, must be performed.

## Conclusion

The cytokine profiles expressed in cattle infected with the *T. vivax* TvET1 isolate from Ecuador indicated that the adaptive immune response is polarized toward both Th1 and Th2, as supported by the presence of proinflammatory and anti-inflammatory cytokines, as well as the IgG1 and IgG2 subclasses. Despite the significant presence of the proinflammatory cytokines IFN-γ and TNF-α, the expression of the anti-inflammatory cytokine IL-10 may contribute to a balance that promotes parasitemia control while protecting the animal from the inflammatory effects of Th1 cytokines. More studies are needed to capture the individual variability of the response to the infection with *T. vivax.*

The information generated in this study is relevant and contributes to the advancement of knowledge host-pathogen interactions. However, several lines of research involve the immune response, starting with exploring the use of IgG1 as a diagnostic target, such as the active search for immunomodulators of the Th1 and Th2 response, as well as the ability to confer protection from the response induced by these molecules for the subsequent development of effective vaccines against *T. vivax*. Furthermore, this study emphasizes the necessity for these new vaccines to trigger an improved IgG2-mediated response. While some studies are already underway in this area, an effective formulation that provides a sufficient level of protection in animals has not yet been achieved.

## Supporting information

S1 Graphical abstract. (TIF)
