# Supplementary figures and images for: Trypanosoma vivax elicits both Th1 and Th2 immunological responses in experimentally infected cattle
Source: PLoS One. 2025 Jul 31;20(7):e0329459. doi: 10.1371/journal.pone.0329459 (PMC12312933; doi:10.1371/journal.pone.0329459)

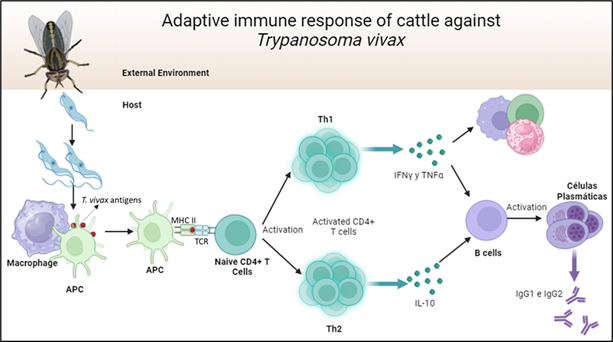

Supplement: S1 — (TIF) [file pone.0329459.s001.tif]
